# Generation of mice with a conditional *Foxp2* null allele

**DOI:** 10.1002/dvg.20305

**Published:** 2007-07

**Authors:** Catherine A French, Matthias Groszer, Christopher Preece, Anne-Marie Coupe, Klaus Rajewsky, Simon E Fisher

**Affiliations:** 1 The Wellcome Trust Centre for Human Genetics, University of OxfordOxford, United Kingdom; 2 The CBR Institute for Biomedical Research, Harvard Medical SchoolBoston, Massachusetts

**Keywords:** *Foxp2*, conditional null allele, Sox2-Cre, speech and language, brain development

## Abstract

Disruptions of the human *FOXP2* gene cause problems with articulation of complex speech sounds, accompanied by impairment in many aspects of language ability. The FOXP2/Foxp2 transcription factor is highly similar in humans and mice, and shows a complex conserved expression pattern, with high levels in neuronal subpopulations of the cortex, striatum, thalamus, and cerebellum. In the present study we generated mice in which *lox*P sites flank exons 12–14 of *Foxp2*; these exons encode the DNA-binding motif, a key functional domain. We demonstrate that early global Cre-mediated recombination yields a null allele, as shown by loss of the *lox*P-flanked exons at the RNA level and an absence of Foxp2 protein. Homozygous null mice display severe motor impairment, cerebellar abnormalities and early postnatal lethality, consistent with other Foxp2 mutants. When crossed to transgenic lines expressing Cre protein in a spatially and/or temporally controlled manner, these conditional mice will provide new insights into the contributions of Foxp2 to distinct neural circuits, and allow dissection of roles during development and in the mature brain. genesis 45:440–446, 2007. Published 2007 Wiley-Liss, Inc.

There are now multiple examples of disruption of the human *FOXP2* gene, with affected individuals displaying speech and language deficits of varying severity (Lai *et al.*, 2001; MacDermot *et al.*, 2005; Shriberg *et al.*, 2006; Zeesman *et al.*, 2006). The first example to be discovered, and hence the most well studied, is that of the KE family. A heterozygous missense mutation is inherited by affected members (Lai *et al.*, 2001), who have an impaired ability to learn and produce the sequences of coordinated mouth movements necessary for speech; a condition commonly referred to as developmental verbal dyspraxia (Vargha-Khadem *et al.*, 2005). These problems are accompanied by expressive and receptive deficits in oral and written language (Watkins *et al.*, 2002). Functional neuroimaging studies have shown abnormal patterns of brain activation, including underactivation of Broca's area, during language-based tasks (Liegeois *et al.*, 2003). To date, *FOXP2* is the only gene clearly linked to this aspect of neurological function, providing a unique opportunity to study the molecular mechanisms involved (Marcus and Fisher, 2003; Vernes *et al.*, 2006).

The FOXP2 protein belongs to a group of transcription factors characterized by the presence of a forkhead-box (FOX) DNA-binding domain. FOX proteins regulate a diverse variety of processes from early embryogenesis through to adulthood, and have been implicated in disorders of human or mouse development (Carlsson and Mahlapuu, 2002). Several domains have been identified in FOXP2 in addition to the characteristic DNA-binding motif, including polyglutamine tracts, a zinc finger, a leucine zipper, and an acidic C-terminal region (Wang *et al.*, 2003). The FOXP2 amino acid sequence is highly similar across a number of distantly-related vertebrate species; the human and mouse proteins are distinguished by only three amino acid substitutions and a single-residue difference in polyglutamine-tract length (Enard *et al.*, 2002). Moreover, orthologues of FOXP2 show conserved expression in equivalent brain structures in humans, rodents, birds, reptiles, and fish, with notable similarities in sublocalisation (Fisher and Marcus, 2006; Vargha-Khadem *et al.*, 2005). Key expression sites lie within the cortex (pallium in nonmammals), striatum, thalamus, and cerebellum. In the mammalian cortex the gene is mainly expressed in the deepest layers, in the rat striatum it is enriched in striosomes, while hindbrain expression is confined to inferior olives, Purkinje cells, and deep cerebellar nuclei in all species studied thus far (Bonkowsky and Chien, 2005; Ferland *et al.*, 2003; Haesler *et al.*, 2004; Lai *et al.*, 2003; Takahashi *et al.*, 2003; Teramitsu *et al.*, 2004).

These data point to widely conserved functions of FOXP2 orthologues in distributed vertebrate circuits involved in sensory processing, sensorimotor integration, and control of skilled coordinated movements. Analyses of primate sequence variation suggest that the precise roles of FOXP2 may have undergone modifications during human history, perhaps in relation to speech (Enard *et al.*, 2002). Nevertheless, animal models will yield crucial insights into the contributions of the gene to the development and function of relevant neural circuits, and how they may go awry in cases of disorder. Gene disruption in the mouse is a highly amenable tool for addressing this question. Given the complexities of Foxp2 expression during embryogenesis, postnatal development and adulthood, in both neural (Ferland *et al.*, 2003; Lai *et al.*, 2003) and non-neural tissues (Shu *et al.*, 2001), a strategy that allows spatiotemporal control of gene disruption is particularly valuable. Thus, in the present study, we have generated a mouse line in which critical exons of *Foxp2* are flanked by *lox*P sites (floxed), and demonstrated successful Cre-mediated disruption of the gene.

It is unfeasible to target the entire *Foxp2* locus, since it spans several hundred kilobases. Instead, we chose to insert *loxP* sites around exons 12–14, which encode the DNA-binding motif (Fig. 1). In addition to removing an essential functional domain, loss of these exons is predicted to yield premature termination of protein translation early in exon 15, because of a frameshift. Of note, Shu et al. previously targeted a similar region of *Foxp2* in standard knockout experiments, and reported that replacement of exons 12 and 13 by a neomycin resistance cassette produced a null allele (Shu *et al.*, 2005). For the present study, Bruce-4 ES cells (Kontgen *et al.*, 1993), of C57BL/6 origin, were transfected with linearized conditional targeting vector. Clones surviving selection were screened by Southern blotting for appropriate integration of the 50 and 30 loxP sites, and the neomycin cassette (Fig. 2a). Correctly targeted clone 1 cells were injected into C57BL/6 albino blastocysts to obtain a male chimera. Breeding to C57BL/6 albino females yielded germline transmission, and *Foxp2*^floxneo/+^ heterozygotes were identified by coat color and PCR genotyping (Fig. 2b).

**FIG. 1:**
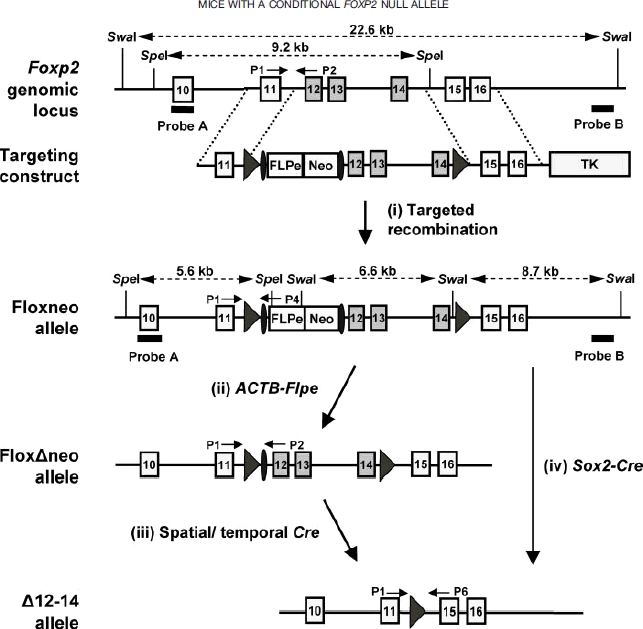
Schematic representation of the *Foxp2* conditional targeting strategy. Recombination of the targeting vector with the *Foxp2* genomic locus results in the introduction of a *FLPe-Neo* cassette, flanked by *FRT* sites (ovals), 5′ of exon 12, and leaves two *loxP* sites (arrows) surrounding the cassette and exons 12–14. The thymidine kinase gene at the 3′ end of the targeting vector enables selection against clones containing randomly integrated vector. FLPe-mediated recombination enables removal of the selection cassette, and generates the *floxδneo* allele. The δ12–14 allele can be generated from either the *floxneo* or *floxδneo* allele by Cre-mediated recombination. PCR genotyping primers and relevant restriction enzyme sites and probes used for Southern analysis are indicated.

**FIG. 2:**
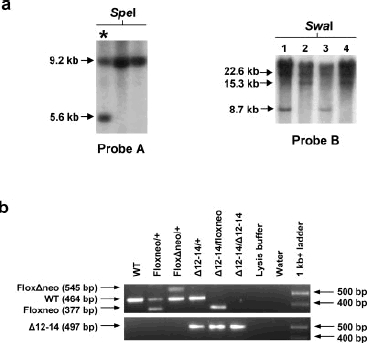
Generation of a *Foxp2* conditional allele. (**a**) Southern analysis of ES cell clone DNA. Left, S*pe*I-digested genomic DNA was hybridized with a probe to exon 10 (Probe A), mapping beyond the 5′ end of the region included in the targeting vector. * marks an example of a correctly targeted clone, demonstrated by the presence of a 5.6 kb fragment. The WT allele gives a 9.2 kb fragment. Right, S*wa*I-digested DNA was hybridized with an intronic probe (Probe B) mapping beyond the 3′ end of the region included in the targeting vector. The WT allele gives a 22.6 kb fragment. Appropriate integration of the 3′ *lox*P site was demonstrated by the presence of an 8.7 kb fragment (clones 1 and 3), whereas recombination within the *lox*P-flanked region yields a 15.3 kb fragment (clones 2 and 4). (**b**) PCR genotyping strategy. Two PCR reactions were used to identify the four *Foxp*2 alleles (cf. Figure 1). The P1/P2/P4 multiplex reaction detects WT, *floxneo* and *floxδneo* (top panel), and the P1/P6 primer pair detects δ*12–14* (bottom panel).

We removed the neomycin resistance gene, since it is well established that the presence of this selection cassette can influence expression of the floxed gene and/or neighboring loci (Lewandoski, 2001). The targeting construct incorporates a *FLPe* gene, driven by the testes-specific *ACE* promoter, which theoretically drives “self-excision” of the *FRT*-flanked selection cassette in the testes. However, we found no evidence that the cassette had been removed in chimeric offspring, probably because of insufficient levels of FLPe protein expression. We therefore mated *Foxp2*^floxneo/+^ heterozygotes to *ACTB-FLPe* hemizygotes [Fig. 1(ii)], which express FLPe ubiquitously under control of human *β-actin* regulatory sequences (Rodriguez *et al.*, 2000) and offspring were genotyped by PCR (Fig 2b). Resulting *Foxp2^floxδneo/+^* heterozygotes were then bred to C57BL/6 wildtype animals to remove the *ACTB-FLPe* transgene from the floxed strain. Mice carrying the *floxδneo* allele will be used for future crosses to transgenic strains expressing Cre recombinase in a region-and/or temporal-specific manner [Fig. 1(iii)].

To verify functionality of the *loxP* sites in vivo, and to determine if Cre-mediated excision of exons 12–14 produces a null allele, we crossed *Foxp2*^*floxneo*/+^ males to *Foxp2*^*δ12–14*/+^; *Sox2-Cre*^+/−^ females [Fig. 1(iv)]. *Sox2-Cre* mice provide an efficient means for deleting *lox*P-flanked sequences, particularly when the *Cre* transgene is carried on the maternal line. In this case excision occurs throughout the early embryo, in all offspring, irrespective of whether they receive the transgene (Hayashi *et al.*, 2003; Vincent and Robertson, 2003). As expected, these crosses produced general deletion of exons 12–14 in offspring and successfully yielded *Foxp2*^*δ12–14*/+^ and *Foxp2*^δ*12–14*/δ*12–14*^ pups, demonstrated by PCR-based genotyping (Fig. 2b). Quantitative real-time RT-PCR was used to analyse *Foxp2* expression in the striatal precursor region of E16.5 embryos from such crosses. Three sets of *Foxp2* primers were employed from different regions of the transcript. *Foxp2*^δ*12–14*/δ*12–14*^ homozygotes lacked the FOX domain-encoding exons – primer pair 13/14 yielded no product in these embryos, and a half-dosage in *Foxp2*^δ*12–14*/+^ heterozygotes (Fig. 3a). RT-PCR assays for exons 6–7 and 16 showed reduced expression levels in *Foxp2*^δ*12–14*/δ*12–14*^ embryos as compared to wildtype littermates, with intermediate levels in *Foxp2*^δ*12–14*/+^ embryos. These data suggest that either a significant proportion of transcripts from the conditional allele are unstable/degraded e.g. by nonsense mediated mRNA decay, or genomic regulatory sequences between exons 12–14 influence expression of *Foxp2*. Of note, mutant mice that carry an early stop codon in exon 7 of *Foxp2* show comparable reductions in *Foxp2* mRNA expression levels (Groszer *et al.*, in preparation). No changes in relative RNA expression were observed for *Foxp1, Foxp4*, or other striatally expressed genes, *Gad2* (Katarova et al., 2000) and *Dlx2* (Porteus et al., 1991) at this developmental time (Fig. 3a).

**FIG. 3:**
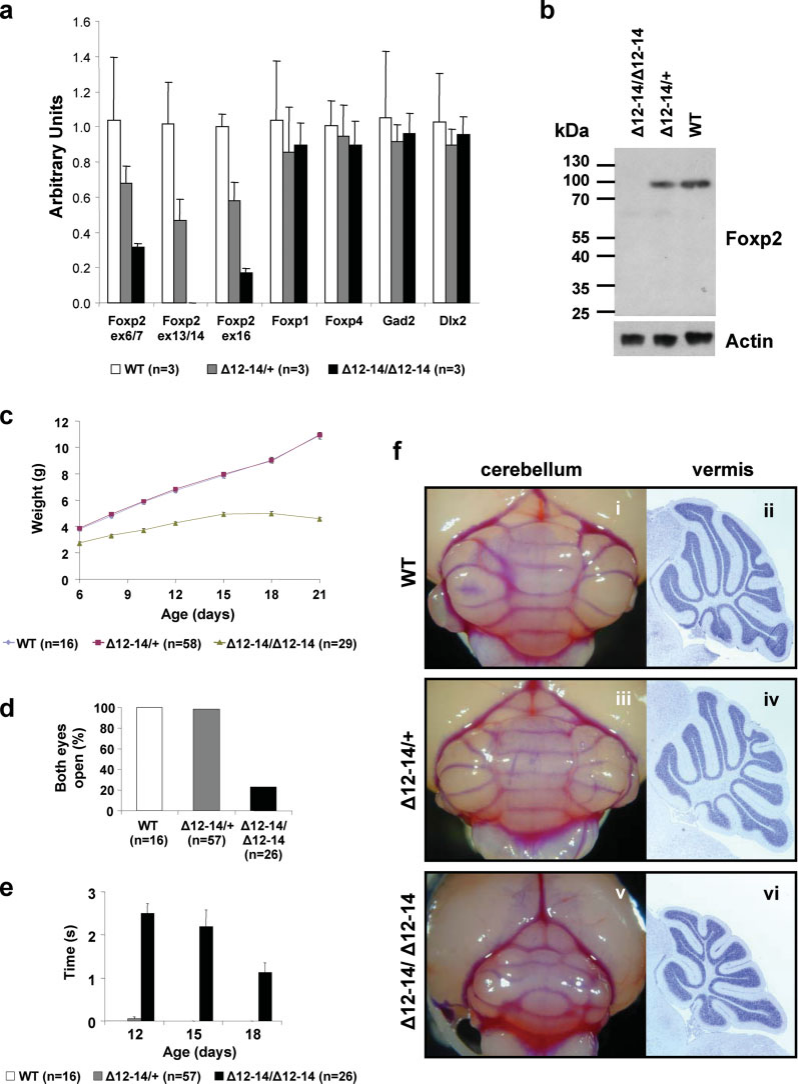
Global Cre-mediated deletion of exons 12–14 yields mice that are null for *Foxp*2. (**a**) Quantitative real-time RT-PCR using RNA from the striatal precusor region of E16.5 embryos (error bars represent SDs). Data confirm a total loss of *Foxp*2 exons 13–14 in transcripts from homozygous mutants, and a half-dosage of these exons in heterozygotes. Flanking exons (6–7 and 16) show reduced expression in mutants. Expression of Foxp1, Foxp4, Gad2, and Dlx2 appears normal in mutants. (b) Western blot analysis of lysates from the striatal precusor region of E16.5 embryos using an antibody recognizing the N-terminus of Foxp2. Reprobing with an anti-actin antibody demonstrates equal protein loading. (**c**) Pup weights (error bars represent SE of the mean). (**d**) Percentage of pups with both eyes open at P15. (**e**) Time taken for pups to right themselves after being placed on their backs (error bars represent SE of the mean); note that WT and heterozygote pups are able to right themselves with virtually no delay. (**f**) Cerebellar morphology at P22 in WT (i, ii), heterozygotes (iii, iv), and homozygotes (v, vi). Homozygotes show reduced cerebellar size and foliation, as shown by whole brains (i, iii, v) and Nisslstained sagittal sections through the vermis (ii, iv, vi). All whole brain photographs were taken at the same magnification.

Extracts from the striatal precursor region of E16.5 embroys were analysed by Western blotting using an N-terminal Foxp2 antibody, to determine the impact of Cre-mediated deletion at the protein level. A single band corresponding to full-length Foxp2 was observed in wildtype extracts and in *Foxp2*^δ*12–14*/+^ extracts at a reduced intensity (Fig. 3b). No bands could be detected in *Foxp2*^δ*12–14*/δ*12–14*^ extracts; there was no evidence of any truncated protein resulting from loss of exons 12–14 and termination of translation in exon 15 (Fig. 3b).

Homozygous *Foxp2*^δ*12–14*/δ*12–14*^ pups have a reduced body weight when compared with littermates, and typically die around postnatal day 21 (Fig. 3c). In addition, they display developmental delays (Fig. 3d) and severe motor dysfunction, including an impaired righting reflex (Fig. 3e). Although most of the brain appears grossly normal,there is a substantial reduction in the size and foliation of the cerebellum (Fig. 3f). By contrast, heterozygous *Foxp2*^δ*12–14*/+^ pups display no overt abnormalities—they gain weight at the same rate as their wildtype littermates, do not show significant righting deficits, and have normal cerebellar size and foliation (Fig. 3c–f). Phenotypes of these homozygous and heterozygous animals are highly consistent with those observed in mutant mice carrying an early stop codon in exon 7 of *Foxp2* (Groszer et al., in preparation). The gross phenotype of *Foxp2*^δ*12–14*/δ*12–14*^ homozygotes also recapitulates that obtained by Shu *et al.* for their standard targeted knockout of exons 12–13 (Shu *et al.*, 2005). However, heterozygotes in the Shu *et al.* study displayed modest developmental delay and reduced rate of weight gain (Shu *et al.*, 2005), unlike our *Foxp2*^δ*12–14*/+^ heterozygotes, which show no such deficits.

In conclusion, we have generated a conditional *Foxp2* allele and shown that homozygous general deletion of exons 12–14 results in absence of Foxp2 protein, accompanied by developmental delays, severe motor dysfunction, and neural abnormalities. Crossing our floxed line with transgenic mice which express Cre-recombinase in a spatially and/or temporally controlled manner should circumvent problems associated with early postnatal lethality and allow investigations of adult animals. Such studies will facilitate investigations of Foxp2 function in the various tissues where it is expressed, including brain, lung, intestine, and cardiovascular system. Most importantly, our conditional allele represents a powerful tool for dissecting the differential contributions of Foxp2 to development and function of distinct neural networks in the mammalian central nervous system.

## MATERIALS AND METHODS

### Gene Targeting and Generation of Mutant Mice

Exon numbering for murine *Foxp2* is concordant with that found for orthologous exons in human *FOXP2* (Mac-Dermot *et al.*, 2005), C57BL/6 DNA containing exons 11–16 of *Foxp2* was cloned into the vector pEASY-FLIRT (Casola, 2004). The resulting construct contained a *loxPFRT-FLPe-Neo^R^-FRT* cassette between exons 11 and 12, with a second *loxP* site inserted between exons 14 and 15. Bruce-4 ES cells were cultured on a mitotically inactive primary MEF feeder layer as described previously (Torres and Kuhn, 1997). 1.2 × 10^7^ ES cells were transfected by electroporation with 25 lg of *Cla*I-linearized targeting construct. Colonies surviving G418 (163 lg/mL active ingredient) and gancyclovir (2 lM) selection were screened for targeted recombination by Southern blot analysis. Morula-stage embryos were harvested 2.5 days post coitum from superovulated C57BL/6 albino females (C57BL/6J-*Tyr^c-2J^*, Jackson Laboratories) and cultured overnight in M16 media. The following day, healthy blastocysts were injected with ES cells and transferred to pseudo-pregnant CD-1 females. Resulting chimeras were bred to C57BL/6 albino mice, to enable germline transmission to be determined by coat color. All regulated procedures were carried out under UK Home Office Project Licence 30/2016.

### PCR Genotyping of Embryos and Mice

Genotyping was performed using lysates prepared from a mouse ear-punch or a small piece of embryo tail. Tissue was digested in 100 μL lysis buffer (50 mM Tris-HCl pH 8.5, 1 mM EDTA, 0.5% Tween 20, 0.5 μg/mL Proteinase K) for 1–2 h at 568C, followed by Proteinase K inactivation at 95°C for 5–10 min. Digested samples were microcentrifuged at full speed for 5 min, and 1 lL of the resulting supernatant was added to a 24 μL PCR mix containing HotStarTaq polymerase (QIAGEN) prepared according to the manufacturer's protocol. The strategy used to genotype the various *Foxp2* alleles is described in Figures 1 and 2 and used the following primers; P1: 5′- TGTCACGTGTGTAAAAAGTCATCTT-3′, P2: 5′-GAGCAT GACAGTGGAATTGAATTAT-3′, P4: 5′-GTCCACTTGTCC CTCACTAGTAAAA-3′, P6: 5′-GGATTAACTATTTCTGGA ATGCAAA-3′. The *Cre* and *FLPe* transgenes were genotyped using primers; Cre1s: 5′-TGATGGACATGTTCAG GGATC-3′, Cre2s: 5′-CAGCCACCAGCTTGCATGA-3′, *Flp1*: 5′-GTGGATCGATCCTACCCCTTGCG-3′, *Flp2*: 5′-GGTCCAACTGCAGCCCAAGCTTCC-3′, yielding fragment sizes of ∼880 and 750 bp respectively. Identical cycling conditions were used for P1/P2/P4 and P1/P6 PCR assays; initial denaturation (95°C for 10 min), product amplification (13 cycles at 95°C for 30 s, 65°C (−0.5°C/ cycle) for 30 s, 72°C for 45 s, followed by 25 cycles at 95°C for 30 s, 58°C for 30 s, 72°C for 40 s), and final extension (72°C for 7 min). *Cre* assays used the following conditions; initial denaturation (95°C for 15 min), product amplification (35 cycles at 94°C for 1 min, 55°C for 1 min, 728C for 1 min), and final extension (72°C for 10 min). FLPe assays used the same conditions as Cre assays except that the annealing temperature was raised to 70°C. DNA fragments were separated by electrophoresis through 1.5% agarose gels.

### Western Blotting

Tissue from the striatal precursor region of E16.5 embryos was dissected into RIPA lysis buffer and disrupted by sonication. Lysates were centrifuged at 10,000g for 10 min, and the protein concentrations of the supernatants were determined by Bradford assay. Proteins (30 μg per lane) were separated on 12% SDS-polyacrylamide gels and transferred to PVDF membranes. Membranes were blocked in 5% milk, before overnight incubation with the primary antibody at 4°C. Goat anti-Foxp2 (N-16) polyclonal antibody (Santa Cruz Biotechnology), and the loading control, mouse antiactin monoclonal antibody (Sigma), were both used at a 1:2,000 dilution. Rabbit anti-goat (Dako) and goat anti-mouse (BioRad) HRP-conjugated secondary antibodies were applied at a 1:5,000 dilution for 1 h at room temperature. Proteins were visualized by chemiluminescence detection using LumiGLO (Cell Signaling Technology).

### Quantitative Real Time RT-PCR

E16.5 striatal tissue, from three brains of each genotype, was dissected into RNA*later* (Ambion), before being snap-frozen and stored at −80°C. When required, samples were thawed and transferred to buffer RLT (QIAGEN) with β-mercaptoethanol, and then disrupted using a piston homogenizer. RNA was extracted using an RNeasy kit (QIAGEN), and included an on-column DNase digestion step. cDNA was synthesized from 2 μg RNA using Superscript III reverse transcriptase (Invitrogen), according to the manufacturer's protocol. PCR amplification was carried out using 25 μL reaction volumes with 12.5 μL of SYBR Green Supermix (BIO-RAD), 0.5 μL of each primer (10 μM) and 1 μL of cDNA. Thermal cycling was performed on the iCycler iQ system (BIO-RAD) with amplification for 50 cycles at 95°C for 15 s, 60°C for 30 s, and 72°C for 30 s. Melting curve analysis was performed to exclude amplification of nonspecific products. Relative changes in expression were calculated using the 2 ^−δδC^T method (Livak and Schmittgen, 2001), using *GAPDH* as the internal control and the average of the wild type samples for each primer pair as the calibrator.

### Histological Analysis

Mice were deeply anaesthetized and transcardially perfused with 4% paraformaldehyde (PFA) in phosphate buffer. Brains were removed and postfixed for a further 24 h, before being embedded in paraffin. Serial sections were cut sagittally at 5 lM and stained with cresyl violet. Whole brains were snap-frozen in liquid nitrogen before being postfixed for 3 h in 4% PFA. Brains were photographed with a drop of bromophenol blue for enhanced contrast.

## References

[b1] Bonkowsky JL, Chien CB (2005). Molecular cloning and developmental expression of foxP2 in zebrafish. Dev Dyn.

[b2] Carlsson P, Mahlapuu M (2002). Forkhead transcription factors: Key players in development and metabolism. Dev Biol.

[b3] Casola S, Gu H, Rajewsky K (2004). Conditional gene mutagenesis in B-lineage cells. B cell protocols.

[b4] Enard W, Przeworski M, Fisher SE, Lai CS, Wiebe V, Kitano T, Monaco AP, Paabo S (2002). Molecular evolution of FOXP2, a gene involved in speech and language. Nature.

[b5] Ferland RJ, Cherry TJ, Preware PO, Morrisey EE, Walsh CA (2003). Characterization of Foxp2 and Foxp1 mRNA and protein in the developing and mature brain. J Comp Neurol.

[b6] Fisher SE, Marcus GF (2006). The eloquent ape: Genes, brains and the evolution of language. Nat Rev Genet.

[b7] Haesler S, Wada K, Nshdejan A, Morrisey EE, Lints T, Jarvis ED, Scharff C (2004). FoxP2 expression in avian vocal learners and non-learners. J Neurosci.

[b8] Hayashi S, Tenzen T, McMahon AP (2003). Maternal inheritance of Cre activity in a Sox2Cre deleter strain. Genesis.

[b9] Katarova Z, Sekerkova G, Prodan S, Mugnaini E, Szabo G (2000). Domain-restricted expression of two glutamic acid decarboxylase genes in midgestation mouse embryos. J Comp Neurol.

[b10] Kontgen F, Suss G, Stewart C, Steinmetz M, Bluethmann H (1993). Targeted disruption of the MHC class II Aa gene in C57BL/6 mice. Int Immunol.

[b11] Lai CS, Fisher SE, Hurst JA, Vargha-Khadem F, Monaco AP (2001). A forkhead-domain gene is mutated in a severe speech and language disorder. Nature.

[b12] Lai CS, Gerrelli D, Monaco AP, Fisher SE, Copp AJ (2003). FOXP2 expression during brain development coincides with adult sites of pathology in a severe speech and language disorder. Brain.

[b13] Lewandoski M (2001). Conditional control of gene expression in the mouse. Nat Rev Genet.

[b14] Liegeois F, Baldeweg T, Connelly A, Gadian DG, Mishkin M, Vargha-Khadem F (2003). Language fMRI abnormalities associated with FOXP2 gene mutation. Nat Neurosci.

[b15] Livak KJ, Schmittgen TD (2001). Analysis of relative gene expression data using real-time quantitative PCR and the 2(-ΔΔ C(T)) method. Methods.

[b16] MacDermot KD, Bonora E, Sykes N, Coupe AM, Lai CS, Vernes SC, Vargha-Khadem F, McKenzie F, Smith RL, Monaco AP, Fisher SE (2005). Identification of FOXP2 truncation as a novel cause of developmental speech and language deficits. Am J Hum Genet.

[b17] Marcus GF, Fisher SE (2003). FOXP2 in focus: What can genes tell us about speech and language?. Trends Cogn Sci.

[b18] Porteus MH, Bulfone A, Ciaranello RD, Rubenstein JL (1991). Isolation and characterization of a novel cDNA clone encoding a homeodomain that is developmentally regulated in the ventral forebrain. Neuron.

[b19] Rodriguez CI, Buchholz F, Galloway J, Sequerra R, Kasper J, Ayala R, Stewart AF, Dymecki SM (2000). High-efficiency deleter mice show that FLPe is an alternative to Cre-loxP. Nat Genet.

[b20] Shriberg LD, Ballard KJ, Tomblin JB, Duffy JR, Odell KH, Williams CA (2006). Speech, prosody, and voice characteristics of a mother and daughter with a 7;13 translocation affecting FOXP2. J Speech Lang Hear Res.

[b21] Shu W, Cho JY, Jiang Y, Zhang M, Weisz D, Elder GA, Schmeidler J, De Gasperi R, Sosa MA, Rabidou D, Santucci AC, Perl D, Morrisey E, Buxbaum JD (2005). Altered ultrasonic vocalization in mice with a disruption in the Foxp2 gene. Proc Natl Acad Sci USA.

[b22] Shu W, Yang H, Zhang L, Lu MM, Morrisey EE (2001). Characterization of a new subfamily of winged-helix/forkhead (Fox) genes that are expressed in the lung and act as transcriptional repressors. J Biol Chem.

[b23] Takahashi K, Liu FC, Hirokawa K, Takahashi H (2003). Expression of Foxp2, a gene involved in speech and language, in the developing and adult striatum. J Neurosci Res.

[b24] Teramitsu I, Kudo LC, London SE, Geschwind DH, White SA (2004). Parallel FoxP1 and FoxP2 expression in songbird and human brain predicts functional interaction. J Neurosci.

[b25] Torres R, Kuhn R (1997). Laboratory protocols for conditional gene targeting.

[b26] Vargha-Khadem F, Gadian DG, Copp A, Mishkin M (2005). FOXP2 and the neuroanatomy of speech and language. Nat Rev Neurosci.

[b27] Vernes SC, Nicod J, Elahi FM, Coventry JA, Kenny N, Coupe AM, Bird LE, Davies KE, Fisher SE (2006). Functional genetic analysis of mutations implicated in a human speech and language disorder. Hum Mol Genet.

[b28] Vincent SD, Robertson EJ (2003). Highly efficient transgene-independent recombination directed by a maternally derived SOX2CRE transgene. Genesis.

[b29] Wang B, Lin D, Li C, Tucker P (2003). Multiple domains define the expression and regulatory properties of Foxp1 forkhead transcriptional repressors. J Biol Chem.

[b30] Watkins KE, Dronkers NF, Vargha-Khadem F (2002). Behavioural analysis of an inherited speech and language disorder: Comparison with acquired aphasia. Brain.

[b31] Zeesman S, Nowaczyk MJ, Teshima I, Roberts W, Cardy JO, Brian J, Senman L, Feuk L, Osborne LR, Scherer SW (2006). Speech and language impairment and oromotor dyspraxia due to deletion of 7q31 that involves FOXP2. Am J Med Genet A.

